# Unveiling the Impact of Antibiotics and Alternative Methods for Animal Husbandry: A Review

**DOI:** 10.3390/antibiotics10050578

**Published:** 2021-05-13

**Authors:** Chuen Xian Low, Loh Teng-Hern Tan, Nurul-Syakima Ab Mutalib, Priyia Pusparajah, Bey-Hing Goh, Kok-Gan Chan, Vengadesh Letchumanan, Learn-Han Lee

**Affiliations:** 1 Novel Bacteria and Drug Discovery (NBDD) Research Group, Microbiome and Bioresource Research Strength (MBRS), Jeffrey Cheah School of Medicine and Health Sciences, Monash University Malaysia, Selangor Darul Ehsan 47500, Malaysia; kelvinlcx@gmail.com (C.X.L.); loh.teng.hern@monash.edu (L.T.-H.T.); syakima@ppukm.ukm.edu.my (N.-S.A.M.); priyia.pusparajah@monash.edu (P.P.); 2 Clinical School Johor Bahru, Jeffrey Cheah School of Medicine and Health Sciences, Monash University Malaysia, Johor Bahru 80100, Malaysia; 3 UKM Medical Molecular Biology Institute (UMBI), UKM Medical Centre, Universiti Kebangsaan Malaysia, Kuala Lumpur 50603, Malaysia; 4 Biofunctional Molecule Exploratory Research Group (BMEX), School of Pharmacy, Monash University Malaysia, Selangor Darul Ehsan 47500, Malaysia; goh.bey.hing@monash.edu; 5 College of Pharmaceutical Sciences, Zhenjiang University, Hangzhou 310058, China; 6 Division of Genetics and Molecular Biology, Institute of Biological Sciences, Faculty of Science, University of Malaya, Kuala Lumpur 50603, Malaysia; 7 International Genome Centre, Jiangsu University, Zhenjiang 212013, China

**Keywords:** antibiotic, multidrug-resistant, growth-promoting, livestock, husbandry

## Abstract

Since the 1950s, antibiotics have been used in the field of animal husbandry for growth promotion, therapy and disease prophylaxis. It is estimated that up to 80% of the antibiotics produced by the pharmaceutical industries are used in food production. Most of the antibiotics are used as feed additives at sub-therapeutic levels to promote growth. However, studies show the indiscriminate use of antibiotics has led to the emergence of multidrug-resistant pathogens that threaten both animal health and human health, including vancomycin-resistant *Enterococcus* (VRE), Methicillin-resistant *Staphylococcus aureus* (MRSA) and carbapenem-resistant *Enterobacteriaceae* (CRE). This scenario is further complicated by the slow progress in achieving scientific breakthroughs in uncovering novel antibiotics following the 1960s. Most of the pharmaceutical industries have long diverted research funds away from the field of antibiotic discovery to more lucrative areas of drug development. If this situation is allowed to continue, humans will return to the pre-antibiotics era and potentially succumb to huge health and economic consequences. Fortunately, studies investigating various alternatives to antibiotics use in livestock show promising results. These alternatives include the application of bacteriophages and phage derived peptidoglycan degrading enzymes, engineered peptides, egg yolk antibodies, probiotics, prebiotics and synbiotics, as well as quorum quenching molecules. Therefore, this review aims to discuss the use of growth-promoting antibiotics and their impact on livestock and provide insights on the alternative approaches for animal husbandry.

## 1. Introduction

The discovery of penicillin by Alexander Fleming in 1928 hailed the coming of a new era in the fight against microorganisms. In the beginning, antibiotics were administered exclusively to humans as a means to combat fatal diseases. In the 1940s, during a time of rising population demand for meat and poultry, extensive research efforts in animal nutrition and feed science were conducted to increase meat production [1]. A study by Stokstad, et al. [2], which was initially designed to investigate the fermentation by-products of *Streptomyces aureofaciens* as an inexpensive source of vitamin B12 for animal feed, discovered that an unknown ingredient in the fermented “mash” greatly increased the growth rate of chickens [2]. The scientists conducted further research and found that this mysterious component was chlortetracycline (Aureomycin), an antibiotic produced by *Streptomyces aureofaciens*. Additional studies later revealed that the addition of small quantities of antibiotics in animal feed was sufficient to promote growth and prevent diseases in chickens and others as well, including dairy cows, turkeys and pigs [3]. These discoveries gave birth to the commercial use of antibiotics as growth promoters in the field of animal husbandry.

**Figure 1:**
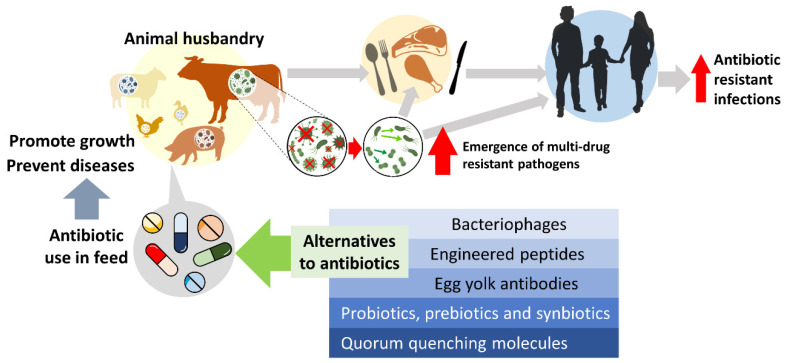
The effect of growth-promoting antibiotics usage in animal husbandry and the potential alternatives for livestock in disease prevention and growth promotion.

In 1951, the Food and Drug Administration in the United States provided the approval for farmers to use antibiotics as a feed additive without the need for a veterinary prescription [4]. Subsequently, between 1950–1970, each European state passed its own regulations to allow the use of antibiotics in livestock feeds. The period coincided with the golden era of antibiotic discovery when scientists discovered the majority of antibiotics that we currently use today [5]. The demand for the use of antibiotics in agriculture flourished due to the lower cost of commercially available antibiotics and growing global consumption of meat. However, the uncontrolled use of antibiotics has given rise to the development of antibiotic-resistant pathogens that could potentially transfer from animals to humans [6,7,8]. Hence, this review aims to discuss the use of growth-promoting antibiotics and their impact on livestock, as well as discuss the potential alternatives methods for animal husbandry (Figure 1).

## 2. How Antibiotics Function as Growth Promoters

Several mechanisms have been proposed as possible explanations on how subtherapeutic levels of antibiotics improved growth in livestock. Gaskins, et al. [9] suggested that administration of sub-therapeutic levels of antibiotics allows animals to reduce their energy spent on maintaining their gastrointestinal commensal bacteria, thereby increasing the overall energy available for growth. This claim is supported by the findings that germ-free chickens, which were raised in isolation, did not experience enhanced growth when fed antibiotics [10]. Under normal circumstances, intestinal bacteria inhabit the intestinal tract of a host, and influence important physiological, immunological and nutritional factors, which maintain the overall health of the host [11,12]. These intestinal bacteria help animals to gain increased protection against colonisation by pathogenic bacteria, possess larger gut size, demonstrate thicker gut walls and display higher numbers of intestinal villi than that of germ-free animals [10]. Unfortunately, the bacteria also absorb nutrients, excrete metabolites, increase gut epithelial turnover and decrease fat digestibility [13]. This can lead to small intestine bacterial overgrowth, which is associated with malabsorption, weight loss and poor health in the animals and affect their growth.

Even at subtherapeutic levels, it was postulated that antibiotics inhibit pathogenic bacterial growth during the animal’s growth phase, which improved their overall health and weight gain [14]. Antibiotics may work by reducing growth—suppressing metabolites, such as bile degradation products, through alterations in the level of bile acid-transforming enzymes—cholyltaurine hydrolase activity in the gut, which leads to increased weight gain in the animals [15]. Antibiotics improve gut barrier function by tamping down wall inflammation and improving nutrient absorption [16]. It is a well-established idea that antibiotics have an anti-inflammatory effect on inflammatory cells, which supports this theory [17,18]. It remains difficult to ascertain an exact mechanism of action of antibiotic growth promoters. We can conclude that antibiotic growth promoters work by influencing both gut microbiota composition and physiological processes in livestock.

## 3. Transfer of Antibiotic Resistance Genes in Livestock

The emergence of resistant bacteria is a result of the Darwinian process of natural selection, where the fittest bacteria survive and propagate themselves. The sub-therapeutic doses administered for growth promotion in animal feeds frequently create optimum selection pressures for the propagation of both pathogenic and commensal resistant bacteria in the gut of the animals by eliminating competition from non-resistant strains [19]. These organisms develop numerous methods to survive selection pressures created by antibiotics, such as antibiotic molecule deactivation by enzymes, use of efflux pumps, and development of cell wall and ribosomal modification to protect cellular targets against antibiotics [20,21]. The main modes of transmission of resistance genes from widespread antibiotic use in livestock occur either through horizontal or vertical transmission [22]. Vertical transmission occurs when the parent organism transfers its genetic material to its progeny. Horizontal transmission involves the transfer of genes through one of these three processes: transformation, transduction and conjugation.

Conjugation occurs when bacteria, which are in close proximity with one another, transfer genetic material through plasmids. This process may occur between the same or different bacterial species, and the plasmids transferred may harbour antibiotic resistance genes. Farm use of antibiotics creates the perfect stress environments for resistant bacteria in the gut to propagate and transfer resistance genes through conjugation when they come into close contact with each other. There is also strong evidence that this process of conjugation is stimulated by the presence of antibiotics [23,24]. In transduction, a virus accidentally copies host bacteria DNA, and upon infecting other bacteria, transfers that DNA into the new host. The incorporation of foreign DNA into a bacteria through this means can sometimes facilitate the transfer of antibiotic resistance genes [25]. This is supported by the identification of resistance genes in bacteriophages isolated from stool samples of both humans and livestock [26,27]. Natural transformation occurs when a bacterium uptakes environmental single-stranded DNA and integrates it into its chromosomes [28]. Several clinically relevant bacterial pathogens, such as *Acinetobacter*, *Neisseria*, *Haemophilus*, *Staphylococcus*, *Pseudomonas*, and *Streptococcus,* are capable of undergoing transformation to acquire antibiotic resistance genes from environmental DNA [28]. The emergence of these resistant bacteria in livestock is then transferred to humans when humans come into contact with these animals or when contaminated meat is consumed by humans [29].

Aside from directly propagating resistant bacterial strains *in vivo*, the antibiotics administered to livestock are not easily degraded by the animals and continue to create perfect stress environments for environmental bacteria when they are passed out of the body through urine and faeces. For instance, studies from China showed high antibiotic residue levels around the areas involved in animal husbandry activities and that 84% of the total antibiotic excretion into the environment originated from farm animals [30,31]. The extent of antibiotics released is massive, as it is estimated that 2.7 trillion pounds of animal manure, which contains antibiotics and antibiotic-resistant organisms, are generated annually in the United States [32]. Furthermore, these contaminated excrements eventually enter our water supplies and other elements in the food chain, causing further pollution [33]. Potential human pathogens in the environment, when exposed to these antibiotic residues, may acquire and develop antibiotic resistance and eventually cause life-threatening infections in humans.

## 4. Emergence of Antibiotic-Resistant Bacteria in Livestock

Over the years, numerous temporal studies have shown that the time of discovery of resistant bacteria corresponds with the time of introduction of a relative antibiotic in livestock [34]. In 1976, Levy, et al. [35] showed a 5-fold increase in detection of tetracycline-resistant *Escherichia coli* isolates from faecal samples of family members of a chicken farm, compared to their neighbours, five to six months after the farmers started introducing tetracycline in their animal feeds. Six months after cessation of tetracycline usage on the farm, the level of tetracycline-resistant microorganisms detected in the faecal samples of the family members of the farm returned to the level comparable to their neighbours. Since then, being a widespread commensal in the gut of farm animals, *E. coli* has been chosen as the indicator microorganism used for monitoring the antimicrobial resistance trends with Gram-negative spectra in livestock [36,37]. Besides studying the antimicrobial resistance in commensal *E. coli* isolated from faeces or manure of farm animals, a recent study identified that clinical *E. coli* isolates from diseased poultry and livestock are commonly resistant to at least three different classes of antibiotics, particularly towards tetracycline, nalidixic acid, sulfamethoxazole and ampicillin [38].

In the Netherlands, from 1982–1989, quinolone resistance in *Campylobacter jejuni* samples isolated from human stools and poultry products increased from 0–11% and 0–14%, respectively, following the introduction of enrofloxacin for poultry use in 1987 [39]. The authors suggested this correlation because humans acquire *Campylobacter jejuni* infections almost exclusively from contaminated poultry products, while the resistance could not have resulted from the clinical usage of fluoroquinolones in humans as the human-to-human transmission of this infection is very rare. In the UK, a similar pattern was demonstrated. Enrofloxacin was licensed for use in poultry in 1994, and the rate of quinolone resistance in *Campylobacter jejuni* isolated from poultry products rose from 1% to 10% between 1991 and 1997 [40]. In recent years, many reports showed that *Campylobacter* spp. from poultry and pig farms in China had high antibiotic resistance rates, particularly towards fluoroquinolones, tetracyclines and macrolides [41,42,43].

The use of avoparcin, a vancomycin analogue, in many European countries as a feed additive was also attributed to the increase in vancomycin-resistant enterococci, a major medical pathogen, in both healthy humans and farm animals from 1989 to 1993 [44,45]. Ever since avoparcin was banned as a growth promoter in 1997 by European Union, the prevalence of vancomycin-resistant *Enterococcus* has markedly declined in food animals [46]. However, Leinweber, et al. [47] reported the first case of vancomycin-resistant *Enterococcus faecium* in Danish poultry farm in 2018 after the ban on avoparcin use. Furthermore, vancomycin resistance genes were detected in the faeces of pigs from several Danish pig farms, suggesting pig faeces as a potential reservoir for the transfer of antibiotic resistance determinants to zoonotic pathogens [48].

Up till today, it is still a debate whether the emergence and dissemination of antibiotic-resistant bacteria that infect humans is a consequence of intensive use of these antibiotics in the farms [49]. For instance, ciprofloxacin-resistant *Escherichia coli* isolates from both poultry and human samples have been found to share similar molecular profiles, which further suggests that drug-resistant human pathogens can originate from farm animals [50]. On the contrary, a study by Graziani, et al. [51] demonstrated that both ciprofloxacin-susceptible and -resistant *E. coli* strains of avian origin were phylogenetically distinct from the ciprofloxacin-resistant *E. coli* strains from humans. Nevertheless, the role of farm animals in the emergence and dissemination of antibiotic-resistant bacteria to humans remains controversial and elusive. This is because of the complexity of the transmission pathways of the antibiotic resistance genes involved in the spread between livestock-to-human, human-to-human and human-to-livestock [52]. Having said that, a recent meta-analysis suggested that to unravel the complex transmission dynamics of resistant bacteria and their antibiotic resistance determinants between humans and food animals requires much effort by combining the genomic data analysis and epidemiological approaches [49].

## 5. Consequences of Antibiotic Resistance

Shortly after the widespread use of antibiotics as growth promoters in livestock in the 1950s, there were multiple reports on outbreaks due to drug-resistant pathogens in both humans and farm animals across various countries [53,54]. Data from the World Health Organization showed that some of the common resistant bacteria include *Escherichia coli, Klebsiella pneumoniae*, *Salmonella* spp., *Streptococcus pneumoniae* and *Staphylococcus aureus* are, in fact, common pathogens of both humans and livestock [55]. The situation is further complicated by the fact that resistance against a particular antibiotic group can offer cross-resistance to another antibiotic group [56,57]. Today, infections with antibiotic-resistant microorganisms lead to significant healthcare and economic losses, and are associated with extended hospital stays, higher mortality rates and greater risk of complications [58,59,60]. The United States Centre for Disease Control (CDC) estimated that in 2019, more than 2.8 million infections and 35,000 deaths were caused by multidrug-resistant infections in the United States [6]. If no further action is taken to address antimicrobial resistance, it has been projected that the number of deaths due to infections caused by multidrug-resistant bacteria could increase up to 10 million yearly in 2050 globally [61,62]. Subsequently, an estimated 1.1–3.8% losses in the annual gross domestic product would be expected by 2050, and pushing 28 million people towards extreme poverty [63].

In order to treat infections by resistant bugs, alternative antibiotics, which usually have inadequate safety profiles, need to be used. For example, colistin, an antibiotic of last resort, is effective in treating highly resistant *Pseudomonas aeruginosa* and *Acinetobacter baumannii* infections; however, it causes severe renal deterioration and failure in patients [64]. It is even more worrying that the therapeutic use of colistin may not last long, as plasmid-mediated resistance against this drug was discovered in *Escherichia coli* isolates derived from pigs in 2016 [65]. If all of our therapeutic antibiotics are eventually rendered useless in the face of resistant bugs, humanity will return to the pre-antibiotic era, where even minor cuts can become infected and lead to catastrophic consequences [66].

## 6. Use of Antibiotics in Livestock Today

Worldwide meat consumption is on the rise, and according to the 2013 statistics from the United Nations Food and Agriculture Organisation (FAO), the most consumed meat worldwide is pork, at 112 million tonnes annually, followed by poultry, at 104 million tonnes annually [67]. As a means of coping with the rising demand for food, the use of antibiotics as growth promoters and feed enhancers were put into practice. In 2014, as high as 80% of the antibiotics produced and sold in the United States were used in animals to promote growth and prevent infections [68]. However, the uncontrolled application of these antibiotics at subtherapeutic doses in food animals, such as for purposes of growth promotion, has significantly contributed to the escalating antimicrobial resistance [69,70,71].

Today, countries with strict regulation have already banned the use of antibiotics as growth promoters. As early as 2006, the European Union banned the use of all in-feed antibiotics [72]. In 2017, the United States Food and Administration (FDA) imposed new regulations to limit antibiotic use in livestock, where clinically important antibiotics are banned for purposes of growth promotion in animal husbandry [73]. Although many of the world’s top meat-producing countries have banned the use of antibiotics as growth promoters in livestock, countries such as China, Russia and India still allow farmers to use the antibiotic growth promoter in livestock [74]. Hence, it is imperative to implement alternatives to the use of antibiotics in major farm animals to curtail the rising antimicrobial resistance and its impact on human morbidity and mortality.

## 7. Alternative Methods for Animal Husbandry

### 7.1. Bacteriophage Therapy and Phage-Derived Peptidoglycan Degrading Enzymes

Bacteriophages are bacteria-infecting viruses with highly specific target populations [75,76]. The serendipitous discovery of bacteriophages is akin to that of many major scientific breakthroughs. The first documented paper on bacteriophage is attributed to F.W. Twort in 1915. In his attempt to describe a medium that was able to culture viruses, he observed that micrococcus, an accidental growth in his agar culture inoculated with vaccinia virus, demonstrated unusual properties which indicate infection [77]. Initially, this discovery did not garner much interest, and Twort was unable to experiment further on his findings due to financial difficulties. Subsequently, in 1917, bacteriologist Felix d’Herelle, detailed how a filtrable agent, which he correctly deduced to be a virus, was infecting and killing the culture of Shiga dysentery bacillus [78]. His paper galvanised the scientific community’s interest into the application of bacteriophages in controlling bacterial pathogens.

Experimental *in-vivo* studies on the administration of bacteriophage therapies were successful in achieving a consistent reduction of *Campylobacter jejuni* populations in broiler chickens [79,80]. Chinivasagam and collegaues demonstrated the use of phage cocktails to control *Campylobacter* in broiler chicken at the farm. The bacteriophages cocktails that were selected to target *C. jejuni* and *C. coli*. The phages were administrated via oral gavage to 47 day old birds for 24 h prior to slaughter. The researcher found that the phage cocktails were effective at reducing *Campylobacter* levels in the market ready broilers. Nevertheless, there were a few birds in farm B showed a low phage titres, and the authors recommended to increase the treatment for over 24 h to ensure continuous phage replication for biocontrol of *Campylobacter in-vivo* [81]. Richards, et al. [82] carried out a study in broiler chicken to determine the efficacy of a two-phage cocktail against *C. jejuni*. The study revealed a significant reduction in caecal counts of the bacterium after two days of treatment, and without affecting the microbiota of the chicken. The bacteriophage administration is proven to be safe compared to the broad bactericidal effects of antibiotics on the animal gut microbiome.

In the context of *Salmonella*, studies have reported the use of anti-*Salmonella* phage cocktail to reduce *Salmonella* colonisation up to 99.9% in the tonsils, ileum and cecum of pigs [83,84]. Vaz, et al. [85] studied the timing effect of phage cocktail (3 lytic phages) therapy against *S. enteritidis* in broiler chickens. After bacterial inoculations on the day of hatching, the chicks received the phage treatments at two intervals, early (6–10 days) and late (31–35 days). The researchers reported both in vivo trials displayed a lower in intestinal *S.* *enteritidis* counts when compared to the control group and higher efficacy in the late phage application. They concluded that multiple phage therapy could enhance the phage ability to control intestinal *S. enteritidis* colonisation in broilers [85]. Recently, a study assessed the efficacy of a patented phage SalmoFREE^®^ against *Salmonella* in broiler chicken on a commercial farm via the animal drinking water. The phage SalmoFREE^®^ has successfully reduced *Salmonella* counts on day 34 of treatment compared to the control group. SalmoFREE^®^ had no adverse effect on the broiler chickens and the production parameters used [86].

Bacteriophage therapy has also been proven to be effective against colibacillosis and clostridiosis in poultry [86,87]. One study showed that a combination of three lytic phages which were fed to naturally avian pathogenic *Escherichia coli* infected chicken resulted in a decrease in the mortality levels, while another study reported that the mortality rate from *Clostridium perfringens* associated necrotic enteritis decreased by 92% in chickens which were given a multivalent bacteriophage cocktail [88,89]. Aside from its use as an antimicrobial agent, bacteriophage therapy also exhibit growth-promoting effects when given to livestock. This was evident by an increased average daily weight gain and enhanced gut villi morphology in pigs that were fed with bacteriophage cocktail in comparison to the control group [90].

The development of an effective bacteriophage therapy does pose challenges for researchers. It has been demonstrated that pathogenic bacteria in poultry exhibit the ability to undergo genomic rearrangement between various phenotypes as a defence mechanism against the environmental selection pressure posed by bacteriophages, giving rise to bacteriophage-insensitive mutants [91,92]. In addition, naturally occurring bacteriophages in the environment may contain reservoirs of antibiotic-resistant genes (ARG) and mediate the transfer of these genes between bacteria [93,94]. However, this is still a subject of debate among researchers, as recent studies suggested that ARGs were overestimated in phages, as shown by the vast differences in predicted versus known ARGs and matches of genomes to proteins unrelated to antibiotic resistance when exploratory thresholds were utilised by researchers in phage genomic analysis [95,96].

Phage-derived peptidoglycan degrading enzymes, namely the virion-associated lysins (VALs) and endolysins, have also been explored as a potential new class of antibacterial agent [97,98]. Numerous in-vitro studies have proved their effectiveness against multi-drug resistant organisms, such as MRSA and VRE organisms [99,100]. Although it was initially challenging to target Gram-negative organisms due to the presence of an outer membrane surrounding the peptidoglycan wall target, researchers successfully modified endolysins by combining them with a polycationic nonapeptide to produce Artilysins which are bactericidal towards *Pseudomonas aeruginosa* and *Acinetobacter baumanni* species [101]. As for in vivo experiments, mice models were largely utilised, where preliminary results showed the effectiveness of endolysins against pathogenic organisms when given during the early stages of infection [102]. It is anticipated that future research efforts will allow VAL to be used as an antimicrobial agent and explore its potential as a growth promoter. Another interesting finding is that peptidoglycan—degrading enzymes derived from certain bacteriophages are highly thermostable. VAL from the *P. aeruginosa* phiKMV bacteriophage retained > 20% of activity after 2 h at 100 °C, while endolysins from multiple thermophilic *Geobacillus* bacteriophage strains were shown to be effective against strains of *C. perfringens* known to cause necrotic enteritis in the poultry and swine [103,104]. This trait is highly desirable as this allows phage-derived peptidoglycans to remain effective even after factory processing as many of commercial animal feeds undergo heat treatment during production.

### 7.2. Egg Yolk Antibodies (EYA)

Egg yolk antibodies is a form of passive immunity offered by the hen to their offspring. Kemplerer first described the transfer of immunoglobulin against the tetanus toxin from hen to chick in 1893 [105]. This discovery leads to a significant interest in the extraction and utilisation of these antibodies to target specific pathogens for use in both animals and livestock.

Out of the various immunoglobulins produced by chickens, IgY is one of the major antibodies that has garnered significant scientific interest. It is present in significant quantities in the egg yolk instead of the other immunoglobulin types present only in small amounts [106]. It is hypothesised that large scale production of IgY is possible, as an average hen would lay enough eggs to produce 100 g of antibodies per year [107]. This production rate of IgY antibodies can be further increased through the utilisation of hens from high antibody-producing genotypes [108].

There is a wide range of benefits of IgY antibody. Immunoglobulin Y (IgY) and the functional counterpart of mammalian IgG are excellent candidates alternatively to antibiotics for preventive and therapeutic against bacteria and virus [109,110]. IgY is also effective in providing protection against a wide range of gastrointestinal pathogens in humans and animals. The IgY antibodies have no harmful effect, toxic residue, disease resistance in animals, and does not cross-act with the mammalian immune system [111]. These antibodies can also be used in animal supplements [112].

A study on the effectiveness of IgY antibodies administration against *Salmonella* spp. infections in poultry showed mixed results. Although the inhibition of *Salmonella enteritidis* growth was demonstrated *in vitro*, the administration of extracted egg yolk antibodies specific to *S. enteritidis* did not significantly reduce gut colonisation when these antibodies were fed directly to the chickens [107,113]. However, in another study, investigators showed a reduction in the rate of *S. enteritidis* contamination in eggs laid by infected hens that were fed with antibody-containing whole egg powder [114].

Karamzadeh-Dehaghani, et al. [111] studied the effectiveness of IgY antibodies against enterotoxigenic *Escherichia coli* K99 *in vitro*. The results showed specific IgY antibodies had the bacterial inhibiting growth at 200 mg/mL. Vandeputte and colleagues studied the reduction of *Campylobacter jejuni* colonisation in broiler chickens after supplemented with IgY antibodies. The in vivo study resulted in a significant reduction in *C. jejuni* counts in the infected birds compared to control broilers [115]. In summary, the results from these in vitro and in vivo studies demonstrated the effectiveness and potential use of IgY antibodies as a replacement for antibiotics in farms.

One notable challenge of the widespread use of immunoglobulins to replace antibiotics is that the pathogen-specific egg yolk immunoglobulins produced do not confer broad-spectrum protection against the various diseases affected by chickens, as opposed to antibiotics [116]. Manufacturing issues, such as the lack of ideal extraction methods and storage instability of egg yolk immunoglobulins, would need to be addressed before large scale production can commence [117]. It is also estimated that the overall cost of egg yolk immunoglobulins would be higher compared to antibiotics [118].

### 7.3. Engineered Peptides

Antimicrobial peptides are amphipathic cationic peptides that are produced endogenously by plants and animals as part of the innate defence system [119,120]. Examples of antimicrobial peptides discovered include bacteriocins, defensins, tachyplesins, β-defensins, protegrins and insect defensins [121,122]. These antimicrobial peptides possess broad-spectrum activity against both Gram-positive and Gram-negative bacteria, exhibit lower minimal inhibitory concentrations compared to antibiotics and offer synergistic effects to combat multidrug-resistant pathogens when administered with antibiotics [123,124,125].

Numerous studies from China showed that antimicrobial peptides provide growth-enhancing and immune-boosting effects when they are fed to chickens [126,127]. Bacteriocins from various microorganisms have also shown promising results in inhibiting the growth of *Clostridium perfringens*, a major cause of necrotic enteritis in broiler chickens, in both in vivo and in vitro studies [128,129,130]. The use of animal peptides, such as spray-dried plasma, has been shown to increase the growth rate of pigs by improving their immunity [131]. Feeding 5–6% of hydrolysed porcine small intestine, a by-product from the extraction of heparin, to post-weaned pigs for two weeks has also shown an increase in feed intake and growth rate, though the mechanisms of action remain unclear. Some of the challenges of widespread use of peptides include the high production cost, loss of efficacy under physiological salt and serum conditions, and potential toxicity issues [132].

A recent study by Daneshmand, et al. [133] revealed the effects of cLFchimera peptide (a recombinant antimicrobial peptide, AMP) in broiler chickens under necrotic enteritis (NE) challenge. The results showed cLFchimera peptide ameliorated NE-related intestinal lesions, reduced mortality and rehabilitated the jejunal villi morphology in NE challenged birds. While the antibiotic non-selectively reduced the count of bacteria, cLFchimera peptide restored microflora balance in the ileum of challenged broiler chickens. The authors concluded that cLFchimera could be a promising candidate to substitute growth promoter antibiotics in farms [133].

The effectiveness of a novel apidaecin Api-PR_19_ produced by engineered prokaryotic expression bacteria as a substitute for antibiotic growth promoters was studied by Wu, et al. [125]. The results demonstrated that broilers fed with Api-PR_19_ supplementation significantly increase the organ index of the bursa of Fabricius and subtype H9 antibody level in broiler chickens. The antibacterial effect of Api-PR_19_ in vitro and in vivo revealed that Api-PR_19_ inhibited the growth of *Escherichia coli* and *Campylobacter jejuni*, without disturbing the beneficial bacteria and microbiota in broilers [125].

Another interesting study by Li, et al. [134] showed that insect defensins are promising candidates against antibiotic-resistant *Staphylococcus aureus*. A variant designed from insect defensin DLP_4_, ID_13_ exhibited strong antibacterial activity at low MIC values of 4–8 μg/mL to Gram-positive pathogens (*S. aureus, S. epidermidis, S. pneumoniae*, *S. suis*), which were lower than those of DLP_4_, and cytotoxicity of ID_13_ (71.4% viability) was less than that of DLP_4_ (63.8% viability). The authors concluded that ID_13_ could be a promising peptide antibiotic agent for therapeutic applications in farms [134].

In summary, the studies above unveiled the potential of antimicrobial peptides as an alternative to antibiotic growth promoters in animal husbandry. These antimicrobial peptides can be produced by bacteria, insects, amphibians, fishes, plant and mammals, as well as by in vitro microbial fermentation using gene engineering strains [135]. They have natural antibiotics properties, low tendency to develop resistance by bacteria, and does not affect the host microbiota. The antimicrobial peptides should be applied in the animal husbandry, thus reducing the effects of antibiotic growth promoters.

### 7.4. Quorum-Sensing & Quorum Quenching

Quorum sensing refers to the process in which cells communicate by producing and detecting extracellular cell signalling molecules known as autoinducers (AI) and alter their gene expression in response to the cell population density [136,137]. It was first described in 1970 by Nealson, et al. [138] when his research team discovered that the bioluminescent bacteria, *Photobacterium fischeri,* secreted the enzyme luciferase in a huge amount during a period of exponential bacterial growth. Over time, it was discovered that pathogenic bacteria also express virulence factors, biofilm formation and drug-resistant behaviours through the process of quorum sensing [139,140].

Quorum quenching refers to a process in which prokaryotes and eukaryotes disrupt the signals involved in quorum sensing. By utilising quorum sensing inhibitors or quorum quenching enzymes, undesirable traits of bacterial behaviour can be controlled [140]. This novel approach to controlling the growth of bacteria is appealing, as the bacteria are not killed—unlike when they are challenged with antibiotics—and resistance is less likely to develop within the bacteria [140].

N-acyl-L-homoserine lactones (AHLs) are regulatory ligands in many Gram-negative organisms that control the expression of virulence genes through regulatory proteins, which are known as LuxR-type proteins. Several quorum quenching molecules, which target AHLs-dependent quorum sensing, have been extensively studied in recent years. The halogenated furanones, which are derived from marine alga *Delisea pulchra*, have been shown to destabilise LuxR activity [141,142]. The enzymes encoded by the *aiiA* gene present in *Bacillus* sp. 240B1 have also been effective in the inactivation of AHLs [143,144]. Similar AHL-lactonases have also been effective in inhibiting biofilm formation by *Vibrio parahaemolyticus* DAHP1 on a coverslip assay [145]. Furthermore, in vivo studies involving zebrafish showed that fish fed with these enzymes displayed significant protection against *Aeromonas hydrophila* infections [146]. Although there are no such studies conducted on livestock animals, it is anticipated that these molecules would produce similar bacterial inhibiting effects, which would replace the use of antibiotics.

The phosphotriesterase-like lactonase (PLL) family of enzymes are another group of quorum quenching molecules that target AHL-mediated quorum sensing systems, which have garnered significant interest [147,148]. The most notable member of this family is the *SsoPox* enzyme, which was isolated from the hyperthermophilic *Sulfolobus solfataricus* [149]. Enzymes from this family are highly attractive for use in biotechnology, as they are found to be highly tolerant of heat, protease degradation, organic solvents and surfactants [150,151]. It is anticipated that the stability of these molecules allows them to be effectively incorporated into materials such as paints, coatings and polymers, which can be used to line the housing areas of livestock to decrease the exposure of the animals to pathogenic bacteria and reduce the need for antibiotics for prophylactic use [140].

### 7.5. Probiotics, Prebiotics and Synbiotics

It has long been an established practice in animal husbandry and aquaculture to include probiotics, prebiotics and synbiotics into animal feeds to promote growth [152]. As the beneficial effects and excellent safety profile of these additives have been well-documented, it is crucial to create more efficient and cost-effective growth promoters as alternatives to antibiotic growth promoters in animal feed, especially in countries where bans have not yet been implemented.

Probiotics are defined as a live microbial feed supplement that beneficially affects the host animal by improving the intestinal microbial balance [153]. Probiotics are known to stimulate not only the growth of host animals but also inhibit the proliferation of harmful pathogenic bacteria [154]. Examples of probiotics commonly administered to livestock include genus *Bacillus, Enterococcus*, and *Saccharomyces* yeast [155]. The administration of *Bacillus* sp. has been found to reduce *Clostridium* and *Salmonella* populations in poultry significantly [156].

Animals fed with *Bacillus subtilis* demonstrate increased weight gain, improved feeding and enhanced gut nutrient absorption; the quality of meat and eggs produced are also enhanced [157]. Chicks that were fed *Lactobacillus acidophilus* and *Streptococcus faecium* probiotics had a 70% reduction in faecal shedding and 27% in jejunal colonisation of *Campylobacter jejuni* [158]. One of the major challenges of probiotics includes the fear that they may facilitate the transfer of antibiotic-resistant genes as they interact with possible pathogenic bacteria in the gut. *Bacillus subtilis* has been found to harbour genes, such as the *aadD2* gene, *bla* (BCL-1) gene, *cat* (BCL) gene, which confer resistance against aminoglycosides, beta-lactams, macrolides and chloramphenicol [159,160,161].

Prebiotics are non-digestible food ingredients that exert a positive effect through their selective metabolism mechanism as they pass through the intestinal tract [162]. Examples of prebiotics include oligosaccharides, acidifiers, protein hydrolysates, plant extracts and many more. They have been shown to improve immune function, anti-viral activities and proliferate certain intestinal bacteria. The major challenges of prebiotics include gastrointestinal side effects (diarrhoea, bloating) and the high production cost [163].

Synbiotics are preparations containing a combination of probiotics and prebiotics. Broilers fed with a combination of probiotics (*Bacillus licheniformis*, *Bacillus subtilis* and *Clostridium butyricum*) and prebiotics (xylooligosaccharide and yeast cell wall) yielded greater breast mass and lower abdominal fat [164]. Supplementation of feeds with Biomin^®^IMBO (combination of *Enterococcus faecium*, cell wall fragments, fructooligosaccharides and phycophytic substances) significantly increased weight gain in chickens [165]. Numerous studies have also been carried out to investigate the beneficial effects of synbiotics when injected *in-ovo*. It was found that an *in-ovo* injection of a combination of *Lactococcus lactis*, galactooligosaccharides and fructan significantly increased the final body weight of the chickens at day 34 [166]. Potential challenges to the use of synbiotics include the high costs of production [167].

## 8. Conclusions and Future Perspective

In conclusion, the complete removal of antibiotics administration in livestock is impossible at this stage, as it would lead to severe disruptions in worldwide meat production. A feasible solution to this age-old problem would focus on utilising alternative agents which are both effective and economical for disease prevention and growth promotion, while retaining the use of antibiotics in treating animal diseases upon veterinary prescriptions. This is a viable option, as it has been shown that with controlled use of antibiotics in livestock, resistance levels can decrease to original parameters in a particular area.

It is exciting to note that apart from the alternatives to antibiotics highlighted above, there are several cutting-edge technologies that are currently being investigated. The spore-producing properties of *Bacillus* spp. have been explored for use in the field of nanobiotechnology. By altering the genes encoding for the spore-coat proteins in *Bacillus* spp., various peptides have been successfully expressed on the surfaces of the spores produced by the genetically-modified bacteria. Upon ingestion of the bacteria, these peptide-containing spores were produced in vivo, and can be designed to exhibit a huge range of properties, such as the ability to adsorb heavy metals, improve enzymatic activity and confer specific immunity [168,169,170]. This may potentially remove the use of antibiotics as growth promoters and in disease prophylaxis in livestock in the future. Furthermore, there is significant interest in the development of CRISPR-Cas9 gene-editing technology, which offers the possibility to reverse antibiotic resistance in specific pathogenic bacteria [171]. The highly specific nature of CRISPR guide RNAs allows for programmed targeting of certain chromosomal and virulence genes, which negates the need to use broad-spectrum antibiotics to treat diseases in livestock [172]. Moreover, a new method of culturing soil bacteria, unlike the conventional Waxman approach, was discovered recently, which led to the discovery of teixobactin, an antibiotic with a novel mechanism of action [173]. This presents an exciting opportunity for the future discovery of novel antibiotics, as previously uncultivable soil bacteria account for 99% of all bacterial species in the environment [173]. We can then reserve the use of these novel antibiotics for therapeutic usage in clinical settings while maintaining the controlled use of antibiotics as growth promoters in livestock to meet agricultural production demands. However, we must not be overly optimistic, as resistance towards novel antibiotics tends to surface soon after antibiotic introduction for widespread use. Nevertheless, the judicious use of existing antibiotics in livestock is highly warranted at this stage, as alternative methods require time to gain the appropriate licensing from regulatory bodies before they can be introduced into the market.
